# Spectrum of combined respiratory chain defects

**DOI:** 10.1007/s10545-015-9831-y

**Published:** 2015-03-17

**Authors:** Johannes A. Mayr, Tobias B. Haack, Peter Freisinger, Daniela Karall, Christine Makowski, Johannes Koch, René G. Feichtinger, Franz A. Zimmermann, Boris Rolinski, Uwe Ahting, Thomas Meitinger, Holger Prokisch, Wolfgang Sperl

**Affiliations:** 1 Department of Paediatrics, Paracelsus Medical University, SALK Salzburg, Salzburg, 5020 Austria; 2 Institute of Human Genetics, Helmholtz Zentrum München, Neuherberg, 85764 Germany; 3 Institute of Human Genetics, Technische Universität München, Munich, 81675 Germany; 4 Department of Paediatrics, Kreisklinikum Reutlingen, Reutlingen, 72764 Germany; 5 Clinic for Pediatrics I, Inherited Metabolic Disorders, Medical University of Innsbruck, Innsbruck, 6020 Austria; 6 Department of Pediatrics, Technische Universität München, Munich, 80804 Germany; 7 Elblab Zentrum für LaborMedizin, Elblandkliniken, Riesa, 01589 Germany

## Abstract

Inherited disorders of mitochondrial energy metabolism form a large and heterogeneous group of metabolic diseases. More than 250 gene defects have been reported to date and this number continues to grow. Mitochondrial diseases can be grouped into (1) disorders of oxidative phosphorylation (OXPHOS) subunits and their assembly factors, (2) defects of mitochondrial DNA, RNA and protein synthesis, (3) defects in the substrate-generating upstream reactions of OXPHOS, (4) defects in relevant cofactors and (5) defects in mitochondrial homeostasis. Deficiency of more than one respiratory chain enzyme is a common finding. Combined defects are found in 49 % of the known disease-causing genes of mitochondrial energy metabolism and in 57 % of patients with OXPHOS defects identified in our diagnostic centre. Combined defects of complexes I, III, IV and V are typically due to deficiency of mitochondrial DNA replication, RNA metabolism or translation. Defects in cofactors can result in combined defects of various combinations, and defects of mitochondrial homeostasis can result in a generalised decrease of all OXPHOS enzymes. Noteworthy, identification of combined defects can be complicated by different degrees of severity of each affected enzyme. Furthermore, even defects of single respiratory chain enzymes can result in combined defects due to aberrant formation of respiratory chain supercomplexes. Combined OXPHOS defects have a great variety of clinical manifestations in terms of onset, course severity and tissue involvement. They can present as classical encephalomyopathy but also with hepatopathy, nephropathy, haematologic findings and Perrault syndrome in a subset of disorders.

## Introduction

**Fig. 1:**
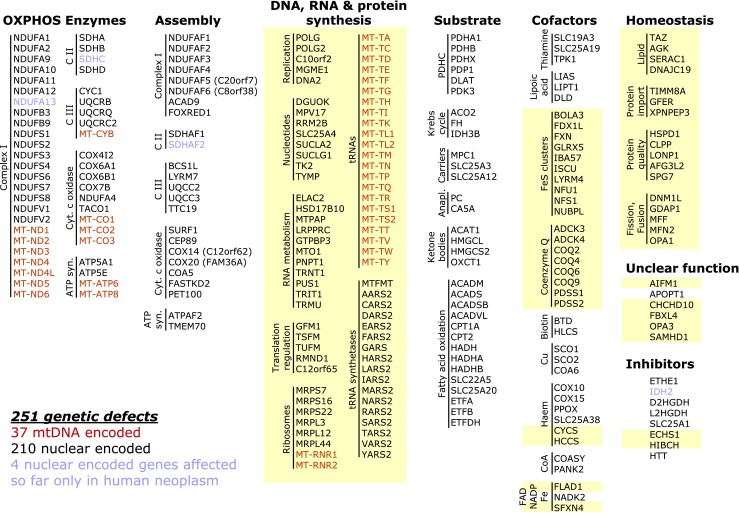
Gene defects (n = 251) of mitochondrial energy metabolism associated with human disease. Gene defects that usually present as combined OXPHOS defects are highlighted in yellow (n = 123)

Mitochondria are cellular organelles essential for aerobic energy metabolism. Proper functioning of mitochondrial energy generation depends on numerous factors. It is assumed that more than 5 % of the human genome plays a role in this metabolism. Indeed, defects involving more than 250 genes (Fig. 1) have been identified to date, making disorders of mitochondrial energy metabolism the most heterogeneous metabolic disease group.

At the heart of mitochondrial energy metabolism is the respiratory chain, which uses the reduced substrates NADH, FADH_2_, ubiquinol and ferrocytochrome c in a step-wise manner to finally react with molecular oxygen to produce H_2_O and establish a proton gradient across the inner mitochondrial membrane. In the final step, the F_1_F_O_-ATP synthase uses the proton gradient to generate the ubiquitous cellular energy carrier ATP. The whole cascade of reactions is called oxidative phosphorylation (OXPHOS).

All five enzyme complexes of OXPHOS consist of multiple protein subunits, in total approximately 75 protein subunits. The complexes depend on several cofactors and the specific lipid environment of the inner mitochondrial membrane to form supramolecular aggregates, which in turn influence the shape of this membrane. Importantly, 13 protein subunits of the OXPHOS enzymes are encoded by the small mitochondrial genome (mtDNA), which is a specific feature of mitochondria and distinguishes them from other cellular organelles in mammalian cells (Schon et al 2012). Although the number of protein-coding genes in mtDNA is small and their functions are limited to OXPHOS, the presence of the mitochondrial genome necessitates having mitochondrial machinery for replication, transcription, RNA processing, RNA modification and translation, complete with its own ribosomes.

## Defects of oxidative phosphorylation

Biochemical investigation of mitochondrial energy metabolism in patient samples dates back to the 1960s, and distinct defects in OXPHOS have been identified affecting either single enzyme complexes or combinations of complexes.

In general, defects of mitochondrial energy metabolism can be grouped into the following five categories (Fig. 1):

1. Isolated defects of OXPHOS subunits or assembly factors.
2. Defects of mitochondrial DNA, RNA and protein synthesis (including replication, nucleotide metabolism, RNA processing and modification and translation).
3. Disorders in the substrate-generating upstream reactions of OXPHOS (pyruvate dehydrogenase complex, Krebs cycle, fatty acid beta-oxidation, substrate import and anaplerosis).
4. Defects in cofactors of OXPHOS and other enzymes of mitochondrial energy metabolism.
5. Defects in the homeostasis of mitochondria, including their biogenesis, lipid processing, protein import, fission/fusion, and quality control.

Furthermore, defects due to inhibition, e.g. by H_2_S in the case of ETHE1 deficiency and inhibition of cytochrome c oxidase (Tiranti et al 2009) or inhibition of mitochondrial protein import by mutated huntingtin (Yano et al 2014), have been reported.

## Combined oxidative phosphorylation defects in diagnostics

Combined OXPHOS defects are a very common finding in the diagnosis of disorders of mitochondrial energy metabolism (Scaglia et al 2004; Gibson et al 2008; Honzik et al 2012). In the patients seen by the diagnostic centre at the Department of Paediatrics in Salzburg, combined OXPHOS defects are by far the most frequent cause of disorders of mitochondrial energy metabolism, with a proportion of 57.3 % (Table 1).

**Table 1:** Number of patients in the diagnostic centre at Salzburg with defects in mitochondrial energy metabolism

OXPHOS defect	Number of patients (% of total)	Genetic diagnoses (%)
Combined defects	177 (57.3 %)	143 (81 %)
Complex I	59 (19.1 %)	30 (51 %)
Complex II	1 (0.3 %)	1 (100 %)
Complex III	7 (2.3 %)	5 (72 %)
Cytochrome c oxidase	28 (9.1 %)	16 (57 %)
ATP synthesis	37 (12.0 %)	35 (95 %)
total	309	230 (74 %)

It is important to point out that combined OXPHOS defects are often picked up in enzymatic measurements as isolated defects. A well-known example is the most frequent m.3243A > G ‘MELAS’ (mitochondrial encephalopathy lactic acidosis and stroke-like episodes) mutation that affects the mitochondrial tRNA^Leu(UUR)^. In muscle biopsies of these patients, an isolated complex I deficiency is a common biochemical finding; however, cytochrome c oxidase-deficient fibres can also be detected (Zierz et al 2014). Other defects are detected mainly as cytochrome c oxidase deficiency (Santorelli et al 1997). Complex I and cytochrome c oxidase seem to be the most vulnerable enzymes. This could be due to their larger number of mitochondrially encoded subunits, especially in the case of complex I (7 subunits, 2117 codons encoded in mtDNA) or cytochrome c oxidase (3 subunits, 1003 codons) versus ATP synthase (2 subunits, 296 codons) and complex III (1 subunit, 380 codons) (Anderson et al 1981). Alternatively, it could be due to different codon distributions; for example, there is a much higher abundance of codons for tRNA^Leu(UUR)^ in ND3 (8.7 % of all codons) and ND6 (9.1 % of all codons) of complex I compared to other mtDNA-encoded proteins, which contain less than 3 % of codons for tRNA^Leu(UUR)^. Finally, the different sensitivities of the OXPHOS complexes might be due to differences in the in vitro assay conditions in different laboratories (Gellerich et al 2004) resulting in experimental bias, since ATP synthesis cannot be quantified in frozen samples.

Therefore, classification as a combined OXPHOS defect in Table 1 was made on the basis of the genetic defect, which was available in 81 % of these patients, in addition to the results of biochemical measurements.

## Typical combinations of defects of oxidative phosphorylation

**Fig. 2:**
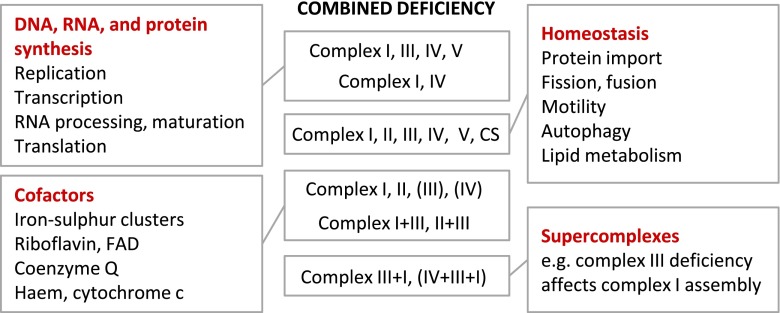
Types of combined respiratory chain defects (typical results) and their causes

By investigation of oxidative phosphorylation enzymes in patient samples, different types of combined defects have been identified: e.g. complex I (CI) + complex IV (CIV), CI + CIII + IV + V, CI + CII + CIII, CI + III/CII + III, CIII + CIV or involvement of all complexes (Fig. 2).

More than one enzyme can be affected due to the following molecular mechanisms:

1. **Mitochondrial DNA-related** (mitochondrial replication, transcription, RNA processing and modification, translation, large deletions of mtDNA)
2. **Cofactor-related** (coenzyme Q, iron-sulphur clusters, haem/cytochromes, riboflavin)
3. **Mitochondrial homeostasis-related** (mitochondrial protein import, lipid metabolism, fission/fusion, mitophagy/quality control)
4. **Supercomplex related** (especially complex III defects)

## Combined defects related to mitochondrial DNA

The mammalian mitochondrial genome is a circular molecule encoding 13 proteins (subunits of complexes I, III, IV and V), two ribosomal RNAs and 22 transfer RNAs. Depending on cell function and size, the number of mitochondria can vary, with copy numbers of mtDNA ranging from just a few to hundreds of thousands per nuclear genome. In contrast to the nuclear genome, mtDNA is replicated in a cell cycle-independent manner. Genetic defects in nuclear genes involved in mtDNA replication, its transcription or translation typically affect only the four OXPHOS enzymes that contain mitochondrially encoded subunits (complexes I, III, IV, and V) but spare complex II and citrate synthase; the latter is commonly used in biochemical analyses as a mitochondrial housekeeping enzyme.

### Defects in mitochondrial replication

Mitochondrial DNA replication seems to require a relatively limited number of proteins (Holt and Jacobs 2014). Up to now, mutations in polymerase gamma (*POLG* gene), its accessory subunit (*POLG2*), and the Twinkle helicase (*C10orf2*) have been reported. Furthermore, mutations of the mitochondrial genome maintenance exonuclease 1 (*MGME1*) (Kornblum et al 2013) and the DNA replication helicase/nuclease 2 (*DNA2*) (Ronchi et al 2013) have been reported to affect mitochondrial stability. The precise role of the latter in mtDNA metabolism is not completely understood and needs further characterisation.

A set of enzymes is required to provide the nucleotides for mtDNA replication: Mutations in the cytosolic enzymes ribonucleotide reductase subunit M2 B (*RRM2B*) and thymidine phosphorylase (*TYMP*) as well as the mitochondrial thymidine kinase (*TK2*), deoxyguanosine kinase (*DGUOK*), succinyl CoA ligase subunit alpha (*SUCLG1*) and beta (*SUCLA2*), an isoform of the adenine nucleotide translocator (*SLC25A4*), and an inner membrane protein of unknown function (*MPV17*) were reported (Copeland 2008).

All of these genetic defects affect mtDNA stability. Accumulation of multiple mtDNA deletions but also point mutations and mtDNA depletion are typical consequences of these nuclear gene defects.

### Defects in mitochondrial transcription, RNA processing and modification

Mitochondrial transcription takes place from both the heavy and light strands to produce a large polycistronic transcript, which has to be processed in order to free the 22 tRNAs, 2 rRNAs and 11 mRNAs, of which two stay polycistronic. Processing takes place predominately at the secondary structures formed by tRNAs. RNase P cleaves at the 5’-end of tRNAs, followed by RNase Z cleavage at the 3’-end. Mutations in HSD17B10, one of the three protein subunits of mitochondrial RNase P, have been shown to result in an increase of unprocessed primary transcripts (Deutschmann et al 2014). Mutations in ELAC2, the mitochondrial RNase Z, lead to an accumulation of mRNAs with tRNAs attached to the 5’-ends (Haack et al 2013). Most mitochondrial mRNAs are modified by MTPAP-mediated polyadenylation; a mutation in this gene resulted in a loss of polyadenylation but remarkably the mRNAs remained oligoadenylated (Crosby et al 2010). Mutations in LRPPRC, a leucine-rich pentatricopeptide repeat-containing protein that is involved in posttranscriptional regulator of mtDNA expression, result in decreased mitochondrial mRNA stability (Sasarman et al 2010). The function of PNPT1, polyribonucleotide nucleotidyltransferase 1, is still a matter of debate, but it might be involved in mitochondrial RNase P RNA import (Wang et al 2012) or part of mitochondrial RNA surveillance (Sarkar and Fisher 2006); mutations result in combined respiratory chain deficiency (Vedrenne et al 2012b).

After processing by RNase Z, the 3’-end of transfer RNAs is modified by addition of CCA, a reaction catalysed by TRNT1. A deficiency of this enzyme affects mitochondrial and cytosolic tRNA modification (Chakraborty et al 2014). Mitochondrial rRNAs, but especially tRNAs, are subject to numerous posttranscriptional modifications catalysed by a battery of enzymes, which are necessary to ensure proper codon–anticodon interaction, folding and stability (Suzuki and Nagao 2011). Mutations affecting tRNA modification have been reported in PUS1 (Bykhovskaya et al 2004), TRMU (Zeharia et al 2009), MTO1 (Ghezzi et al 2012), GTPBP3 (Kopajtich et al 2014) and TRIT1 (Yarham et al 2014), resulting in decreased de novo protein synthesis in mitochondria.

### Defects in mitochondrial translation

Mitochondria have their own ribosomes with at least 80 ribosomal proteins forming the large 39S and small 28S subunits (Rackham and Filipovska 2014). Mutations have been reported in MRPL3 (Galmiche et al 2011), MRPL12 (Serre et al 2013), MRPL44 (Carroll et al 2013), MRPS16 (Miller et al 2004) and MRPS22 (Saada et al 2007) so far. The 12S and 16S ribosomal RNAs are encoded on the mitochondrial DNA. For translation, 22 mitochondrially encoded tRNAs are required, which are hot spots for mutation. More than 250 pathogenic mutations of mitochondrial tRNAs have been identified (Ruiz-Pesini et al 2007). Loading of each tRNA with its proper amino acid necessitates 19 aminoacyl tRNA synthetases, 17 of which are specific for mitochondria and two (GARS, KARS) are shared between the cytosol and mitochondria. With the exception of PARS2 and WARS2, mutations have been reported in all of these aminoacyl tRNA synthases (Diodato et al 2014; Hallmann et al 2014; Schwartzentruber et al 2014; Vanlander et al 2014). Like in bacteria, nascent mitochondrial proteins start with N-formylmethionine, which is generated by methionyl-tRNA formyltransferase (*MTFMT*) using the substrates Met-tRNA^Met^ and 10-formyl-tetrahydrofolate. Mutation of this gene leads to a translation deficiency in mitochondria (Tucker et al 2011). Mitochondrial translation is regulated by several factors, and mutations affecting this process have been found in the translation elongation factors G (*GFM1* gene, (Coenen et al 2004)), Ts (*TSFM* gene, (Smeitink et al 2006)) and Tu (*TUFM* gene, (Valente et al 2007)), in the release factor C12orf65 (Antonicka et al 2010), and in the conserved mitochondrial membrane protein RMND1, whose function in mitochondrial translation is uncharacterised (Garcia-Diaz et al 2012; Janer et al 2012).

### Large deletions of the mitochondrial DNA

The midpoint of single large deletions of mtDNA is usually located between positions 9000 and 13,500, with deletion sizes ranging between 2 and 9 kb (Grady et al 2014). Typically, large deletions cover several protein-coding genes and also affect tRNAs. Large deletions are thereby a classic example of combined OXPHOS defects due to loss of protein-coding genes in combination with tRNA deficiency that results in mitochondrial translation defects.

## Combined defects due to deficiency of cofactors

Numerous cofactors play an essential role in mitochondrial energy metabolism. Some of these cofactors are required for several of the respiratory chain enzymes like coenzyme Q, iron-sulphur clusters, riboflavin and haem. Their deficiency typically results in defects of more than one respiratory enzyme.

### Coenzyme Q deficiency

This cofactor, which is also called ubiquinone, is a lipid compound consisting of a quinone group that can absorb one (forming a semiquinone) or two electrons and a side chain of variable isoprenoid residues. In the case of human ubiquinone, the side chain consists of ten isoprenoid residues (CoQ_10_). CoQ_10_ carries electrons from complex I and complex II to complex III, but is also necessary for other reactions like those catalysed by the electron-transferring-flavoprotein dehydrogenase (ETFDH) (involved in fatty acid oxidation and amino acid catabolism) and the mitochondrial glycerol-3-phosphate dehydrogenase (part of the redox transporting glycerol-3-phosphate shuttle). The synthesis of coenzyme Q takes place in mitochondria, and several defects of coenzyme Q biosynthesis affecting either enzymatic steps (PDSS1, PDSS2, COQ2, COQ6), regulatory proteins (ADCK3, ADCK4, COQ4) or an unknown function (COQ9) have been reported (Desbats et al 2014; Brea-Calvo et al 2015). Typically these defects present with defects of complex I + III and II + III, activities that require coenzyme Q (Lopez et al 2006). In addition to defects in CoQ_10_ synthesis, secondary deficiency has been found in several other genetic disorders: APTX, (Quinzii et al 2005), ETFDH, (Gempel et al 2007), BRAF, (Aeby et al 2007), methylmalonic acidaemia, (Haas et al 2009) and ANO10 (Balreira et al 2014). Supplementation with CoQ_10_ can improve the outcome of several of these defects significantly (Quinzii et al 2014).

### Defects in iron sulphur cluster synthesis

Iron sulphur (FeS) clusters are an ancient compound consisting of varying proportions of iron and sulphur forming an oligomeric molecule with alternating iron and sulphur atoms, in humans predominantly rhombic [2Fe-2S] and cubane [4Fe-4S]. The initial part of FeS cluster biosynthesis takes place in a stepwise process in mitochondria with cysteine as the sulphur donor and iron in the form of a Fe^2+^ ion (Stehling et al 2014). Due to their capability to accept and donate electrons, FeS clusters are involved in numerous redox reactions, including those carried out by respiratory chain complexes I, II and III but also aconitase from the Krebs cycle and lipoic acid synthetase. The latter is necessary for mitochondrial lipoic acid synthesis and therefore essential for 2-ketoacid dehydrogenases like pyruvate dehydrogenase and α-ketoglutarate dehydrogenase (Mayr et al 2014).

Mutations in the early steps of FeS biosynthesis affecting FDX1L, FXN, ISCU, NFS1 or LYRM4 result in deficiency of the respiratory chain complexes I, II, III and aconitase but affect lipoic acid synthesis to a lesser extent. Mutations in BOLA3, NFU1, IBA57 and GLRX5 result in defects of mitochondrial 4Fe-4S synthesis and a pronounced deficiency of complex II, complex I and lipoic acid synthesis but spare complex III and aconitase. Mutations in NUBPL result in isolated complex I deficiency (Stehling et al 2014).

### Other cofactors

Cytochromes (a, a3, b and c) with prosthetic **haem** groups are essential parts of respiratory chain complexes. Deficiencies of haem biosynthesis are known to result in several types of porphyria (Puy et al 2010), but no involvement of the respiratory chain has been reported in these patients. Autosomal dominant mutations have been identified in CYCS, encoding cytochrome c, which carries electrons from complex III to cytochrome c oxidase (Morison et al 2008). Haem c is transferred to apo-cytochrome c by holocytochrome c synthase encoded by the X-chromosomal *HCCS* gene. Mutations have been identified in heterozygous females and in a male with somatic mutations (van Rahden et al 2014). Recently, a mutation of FLAD1, which encodes the **FAD** synthase, was identified by a genetic screening approach in a single patient with combined complex I and cytochrome c oxidase deficiency (Taylor et al 2014). A combined defect of complex I and I + III has been identified in SFXN4 deficiency, affecting an inner membrane protein with a potential role in iron transport (Hildick-Smith et al 2013).

## Defects in mitochondrial homeostasis

Mitochondrial homeostasis involves several essential aspects of mitochondrial biogenesis, lipid synthesis, protein import, fission and fusion, quality control and targeted degradation.

### Defects in mitochondrial lipid synthesis

Mitochondrial membranes consist of a high proportion of non-bilayer forming lipids. De novo synthesis of the phospholipids phosphatidylethanolamine, cardiolipin (CL) and phosphatidylglycerol takes place in mitochondria (Mayr 2014). Defects have been found in tafazzin, which is encoded by the X-chromosomal *TAZ* gene, and cause Barth syndrome (MIM 302060) and combined OXPHOS defects (Karkucinska-Wieckowska et al 2013). Tafazzin is involved in remodelling of CL. Mutations in the mitochondrial co-chaperone DNAJC19 are associated with cardiomyopathy and 3-methylglutaconic aciduria. DNAJC19 acts in a complex with prohibitin (PHB). Absence of this complex leads to the accumulation of CL species with altered acyl chains similar to those in Barth syndrome (Richter-Dennerlein et al 2014). De novo synthesis of CL starts from phosphatidic acid (PA). PA formation from diacylglycerol seems to be limiting in AGK deficiency, which is the genetic cause of Sengers syndrome (MIM 212350) (Mayr et al 2012) with combined deficiency of complex I and the adenine nucleotide translocator (Haghighi et al 2014). An abnormal fatty acid composition of phosphatidylglycerol and decreased bis(monoacylglycero)phosphate (BMP) concentration have been found in patients with MEGDEL syndrome and SERAC1 deficiency (Wortmann et al 2012). Abnormal lipid composition, especially of CL, affects the activity of OXPHOS enzymes and also of adenine nucleotide translocator (Mayr 2014).

### Deficiencies in protein import, processing and quality control

The highly conserved mitochondrial protein import machinery has been mostly unravelled by studies in yeast (Neupert and Herrmann 2007). In stark contrast to the complexity of this system, only a few defects have been identified, including deficiency of the X-chromosomally encoded subunit TIMM8A of the inner membrane translocase associated with Mohr-Tranebjaerg syndrome (MIM 304700) (Jin et al 1996). A deficiency of the disulphide relay system protein GFER is associated with cataract and combined respiratory chain deficiency (Di Fonzo et al 2009).

Defects of the X-prolyl aminopeptidase 3 encoded by *XPNPEP3* result in improper N-terminal protein processing and instability of several subunits of OXPHOS enzymes (Vogtle et al 2009; O'Toole et al 2010).

Protein quality control in mitochondria involves several pathways. Mutations have been identified in the following: *HSPD1*, encoding the conserved heat shock protein 60 (Hansen et al 2002); *CLPP*, a caseinolytic mitochondrial matrix peptidase proteolytic subunit (Jenkinson et al 2012); *SPG7*, encoding a component of the m-AAA protease termed paraplegin (Casari et al 1998); and *AFG3L2*, encoding the catalytic subunit of the m-AAA protease (Cagnoli et al 2006). Defective respiration has been demonstrated in AFG3L2 complementation studies in yeast (Di Bella et al 2010). Multiple deletions of mtDNA have been identified in muscle biopsies of patients with SPG7 deficiency (Pfeffer et al 2014; Wedding et al 2014) and also with AFG3L2 deficiency (Gorman et al 2014).

### Defects of mitochondrial fission and fusion

Mitochondria are dynamic organelles which undergo fission and fusion. The protein machinery needed for fission is widely conserved between mitochondria and peroxisomes (Schrader et al 2012). A central protein in fission is the dynamin 1-like protein DNM1L, frequently also termed Drp1, which forms a ring structure around mitochondria to promote fission. Heterozygous mutations cause deficiency in mitochondrial and peroxisomal fission (Waterham et al 2007). Mutations have also been identified in the mitochondrial fission factors MFF and GDAP1, leading to impaired mitochondrial fission and respiratory chain deficiency (Cassereau et al 2009; Shamseldin et al 2012).

Defects of mitochondrial fusion have been linked to MFN2 (mitofusin 2), a dynamin-like GTPase protein enriched at the endoplasmic reticulum–mitochondria interface, and to OPA1, also a dynamin-like GTPase protein located in the inner mitochondrial membrane and involved in several processes, including mitochondrial fusion. Mutations in these two proteins result in multiple deletions of mtDNA and combined OXPHOS deficiency (Hudson et al 2008; Rouzier et al 2012).

## Combined defects related to genes with unclear mitochondrial function

The precise mitochondrial functions of some proteins that cause combined OXPHOS defects are not yet clear. The X-chromosomally encoded AIFM1, well known as an apoptosis-inducing factor, seems to have a mitochondrial function as an NADH oxidoreductase; however, the association with OXPHOS deficiency is not well understood. Similarly the nature of the cytochrome c oxidase decrease in APOPT1 deficiency, a mitochondrial protein termed apoptogenic 1 and known from apoptosis studies, is not well understood (Melchionda et al 2014). CHCHD10 is a coiled-coil-helix-coiled-coil-helix domain-containing protein of unknown function localised to the intermembrane space of mitochondria, and its deficiency causes multiple deletions of mtDNA and combined OXPHOS deficiency (Bannwarth et al 2014). FBXL4, an F-box and leucine-rich repeat protein, is also an intermembrane space mitochondrial protein of unknown function. Deficiency of FBXL4 causes a decrease of all OXPHOS subunits but also of other mitochondrial proteins and mtDNA (Bonnen et al 2013; Gai et al 2013). Deficiency of the mitochondrial protein OPA3 causes 3-methylglutaconic aciduria, which has been found in several other defects of mitochondrial energy metabolism (Wortmann et al 2013), and fragmentation of the mitochondrial network (Grau et al 2013); however, the precise function of OPA3 remains unclear. Finally, multiple deletions of mtDNA have been reported in one study of a family with Aicardi-Goutieres syndrome 5 (MIM 612952) and SAMHD1 deficiency (Leshinsky-Silver et al 2011). The molecular link of SAMHD1 to mitochondrial DNA is not clear but could be related to its function in deoxynucleotide metabolism.

## Combined defects due to toxic metabolites

Accumulation of highly reactive metabolites like methacrylyl-CoA has been reported in defects of isoleucine catabolism, which takes place in mitochondria. This compound forms covalent bonds, e.g. with the sulphhydryl group of cysteine in proteins, which can destroy enzymes (Brown et al 1982). In fact, combined OXPHOS defects have been reported in HIBCH- (Loupatty et al 2007) and ECHS1- (Sakai et al 2014) deficient patients. In addition to these defects, combined OXPHOS deficiency has been reported in several forms of organic aciduria like propionic acidaemia and methylmalonic acidaemia (de Keyzer et al 2009).

## Supercomplex-related multiple OXPHOS defects

In 2000 Schägger and Pfeiffer (Schagger and Pfeiffer 2000) introduced the concept of a respirasome with oligomerisation of the respiratory chain complexes and formation of domain structures on the inner mitochondrial membrane. In addition, oligomerisation of the ATP synthase has been shown, which is also integral for inner membrane structure (Wittig and Schagger 2008).

Mouse cells harbouring a high mutation load in cytochrome b, a mitochondrially encoded subunit of complex III, have been shown to be deficient in both complex III and complex I (Acin-Perez et al 2004). Homozygous loss-of-function mutations in cytochrome b have been reported in human oncocytic tumours with a complete loss of complex I (Gasparre et al 2008; Zimmermann et al 2011), which is clear evidence that assembled complex III is necessary for complex I assembly and supercomplex formation. Also a mutation in the UQCRC2 subunit resulted in aberrant supercomplex formation and deficiency of complex I in addition to complex III (Miyake et al 2013). Similar results were found in a knockdown cell line of Rieske iron-sulphur protein, another subunit of complex III (Diaz et al 2012). Furthermore, a deficiency of supercomplex formation was shown in SURF1 deficiency, which is known to be an assembly factor of cytochrome c oxidase (Kovarova et al 2012). Defective supercomplex formation (McKenzie et al 2006) and combined OXPHOS deficiency (Karkucinska-Wieckowska et al 2013) have also been found in patients with Barth syndrome and TAZ mutations leading to an increased lysocardiolipin pool in mitochondria.

This summary, although incomplete, demonstrates that defects in single subunits of OXPHOS enzymes and individual assembly factors but also in the lipid composition can result in deficiency of supercomplex formation and hence a combined OXPHOS deficiency.

## Clinical presentation of combined OXPHOS defects

The clinical phenotypes associated with combined OXPHOS defects are very heterogeneous, but in many cases encephalomyopathy is the main presentation. A very well-characterised example is the most common “MELAS” mutation m.3243A > G that can result in different clinical symptoms aside from MELAS, including sensorineural hearing loss, (isolated) myopathy, cardiomyopathy, seizures, migraine, ataxia, cognitive impairment, bowel dysmotility, short stature, diabetes, external ophthalmoplegia and Leigh syndrome (Nesbitt et al 2013). Since this mutation affects the mtDNA, the mutation load is variable and can be different in different tissues. Affected individuals usually carry this mutation in a high proportion; however, clinically unaffected or just mildly affected maternal relatives who carry a high mutation load are also found in these pedigrees (Dubeau et al 2000). Another well-studied example of clinical heterogeneity concerns patients with mutations in the *POLG* gene, encoding mitochondrial DNA polymerase γ. The clinical features of deficiencies in this gene include seizures and hepatopathy (Alpers disease), ataxia, neuropathy, myopathy, chronic progressive external ophthalmoplegia, ptosis, sensorineural deafness, parkinsonism and premature ovarian failure, hypogonadism and gastrointestinal dysmotility (Tchikviladze et al 2014). The same causative mutation can be either autosomal recessive or dominant, the latter usually resulting in delay of disease onset to adulthood.

As illustrated by these two examples, it is not possible to describe a general clinical picture of combined OXPHOS defects. In the following, some clinical features and syndromes are summarised that are associated with certain types of combined OXPHOS and can be helpful in the diagnosis of patients:

**Hepatopathy** is found only in certain defects of mitochondrial energy metabolism but especially in a number of combined OXPHOS disorders (Table 2). Hepatic presentation is frequently encountered in disorders of mitochondrial replication associated with POLG or C10orf2 (Twinkle); in disorders of mitochondrial nucleotide metabolism involving DGUOK, MPV, SUCLG1 and TRMU (usually transient infantile manifestation); in aberrant translation regulation by TSFM (Vedrenne et al 2012a) and in some cases of GFM1 deficiency, and was also reported in patients with EARS2 (1 patient) and FARS2 deficiency (Rahman 2013). Furthermore, hepatopathy is also a relatively common feature in MEGDEL syndrome with SERAC1 deficiency, which involves lipid metabolism (Wortmann et al 1993).

**Table 2:** Characteristic clinical manifestations found in some gene defects associated with combined OXPHOS deficiency

Hepatopathy	Nephropathy	Perrault syndrome	Haematologic manifestation	Leigh-(like)
*POLG*	*RRM2B*	*HARS2*	*PUS1*	*TYMP*
*C10orf2 (Twinkle)*	*C10orf2 (Twinkle)*	*LARS2*	*YARS2*	*MTPAP*
*DGUOK*	*TSFM*	*CLPP*	*TRNT1*	*LRPPRC*
*MPV*	*MRPS22*	*C10orf2 (Twinkle)*	*SFXN4*	*PNPT1*
*SUCLG1*	*SARS2*		*GLRX5*	*TUFM*
*TRMU*	*ADCK4*		*FBXL4 (partially)*	*GFM1*
*EARS2*	*COQ2*		*CYCS*	*C12orf65*
*FARS2*	*COQ6*		*TAZ*	*MT-TI*
*TSFM*	*COQ9*		*MT-TL1*	*MT-TK*
*GFM1*	*PDSS2*		mtDNA deletions	*MT-TL1*
*SERAC1*	*XPNPEP3*			*MT-TV*
	*MT-TF*			*MT-TW*
	*MT-TI*			*MTFMT*
	*MT-TL1*			*PDSS1*
	*MT-TN*			*SERAC1*
	*MT-TY*			*AIFM1*
	mtDNA deletions			

**Nephropathy** may be an underdiagnosed sign of mitochondrial disease but it has been reported in several combined OXPHOS defects. Proximal tubulopathy is a typical finding in early onset mitochondrial DNA depletion syndrome caused by RRM2B deficiency (Bourdon et al 2007) and was also reported in a family with C10orf2 (Twinkle)-deficient patients (Prasad et al 2013). Renal tubulopathy is further found in translational defects involving SARS2, MRPS22 and TSFM (O'Toole 2014). In coenzyme Q synthesis defects, nephrotic syndrome (ADCK4, PDSS2, COQ2, COQ6) and tubulopathy (COQ9) are leading features (Desbats et al 2014). Patients with XPNPEP3 deficiency, encoding X-prolyl aminopeptidase 3, develop a nephronophthisis-like nephropathy but can also involve other organs (O'Toole et al 2010). Furthermore, tubulointerstitial nephritis and focal segmental glomerulosclerosis have been associated with various mitochondrial tRNA mutations, and single deletions of mtDNA have been reported to cause proximal as well as distal tubulopathy (O'Toole 2014).

**Perrault syndrome** is an autosomal recessive disorder characterised by sensorineural hearing loss in males and females and ovarian dysfunction in females. Neurologic features have been described in some affected women (Newman et al 1993). To date, mutations in five genes (CLPP, HARS2, LARS2, C10orf2 [Twinkle] (Morino et al 2014), HSD17B4) have been reported, with all but the last causing combined OXPHOS deficiency.

**Haematologic manifestations** of combined OXPHOS defects include aplastic, macrocytic or sideroblastic anaemia, leukopenia, neutropenia, thrombocytopenia or pancytopenia. **Sideroblastic anaemia** is characterised by the presence of ringed sideroblasts in the bone marrow and can be caused by PUS1 deficiency (affecting mitochondrial pseudouridine synthase) and presenting clinically as myopathy, lactic acidosis and sideroblastic anaemia (MLASA) (Bykhovskaya et al 2004). Deficiency of YARS2, the mitochondrial tyrosyl-tRNA synthetase, also results in a MLASA phenotype (Riley et al 2010). Recently, patients with mutations in TRNT1 (tRNA CCA-adding nucleotidyl transferase) have been reported. Clinically they present with congenital sideroblastic anaemia with immunodeficiency, fever and developmental delay (SIFD) (Chakraborty et al 2014). Two patients with either homozygous (Camaschella et al 2007) or compound heterozygous (Liu et al 2014) mutations in GLRX5, a mitochondrial enzyme of iron sulphur cluster maturation also needed for haem biosynthesis, have been described. **Macrocytic anaemia** with megaloblastic features has been reported in patients with SFXN4 deficiency, an inner mitochondrial membrane protein with a presumed iron transport function (Hildick-Smith et al 2013). **Thrombocytopenia** has been reported as a major feature in patients with autosomal dominant CYCS deficiency of cytochrome c (Morison et al 2008; De Rocco et al 2014). **Neutropenia** is associated in male patients with Barth syndrome, caused by TAZ mutations, in addition to cardiomyopathy, skeletal myopathy, prepubertal growth delay and a distinctive facial gestalt (Ferreira et al 1993). In addition to global developmental delay, hypotonia and other clinical features, neutropenia has been identified in patients deficient in FBXL4, an intermembrane space mitochondrial protein involved in mitochondrial biogenesis (Gai et al 2013). Furthermore, neutropenia has also been reported in a patient with the common m.3243A > G mutation in the MT-TL1 gene (De Kremer et al 2001). Pearson marrow–pancreas syndrome (MIM 557000) is caused by deletions of mtDNA, with variable generalised clinical manifestations, including haematologic presentation with early transfusion-dependent anaemia, neutropenia, thrombocytopenia, and, less abundant, also ringed sideroblasts in bone marrow aspirates (Broomfield et al 2014).

**Leigh syndrome** (LS, MIM 256000) is characterised by progressive neurologic disease with motor and intellectual developmental delay, signs and symptoms of brain stem and/or basal ganglia disease, and raised lactate (Thorburn and Rahman 1993). LS or Leigh-like presentation is most prevalent in defects of OXPHOS subunits and assembly factors, but also several combined OXPHOS defects present with this neurologic manifestation and involve either mitochondrial nucleotide or RNA metabolism, translation, a form of coenzyme Q deficiency, and a few defects in mitochondrial homeostasis (Table 2).

## Conclusion

Combined OXPHOS defects are the most frequent cause of disorders of mitochondrial energy metabolism found in nearly half of the known gene defects (Fig. 1) and affecting more than 50 % of patients (Table 1).

Diagnosis of combined OXPHOS defects is complicated by the fact that the degree of individual OXPHOS enzyme decrease involvement is not necessarily identical among the different gene defects and it can vary between tissues. Therefore, combined OXPHOS defects can be picked up as isolated defects, especially in cases with mild manifestation.

Clinically, combined OXPHOS defects are highly heterogeneous with a broad spectrum of possible signs even for identical mutations (especially of the mtDNA). Several features are restricted to a subset of genetic defects (Table 2), which may help to pinpoint the underlying molecular cause of a combined respiratory chain deficiency.

## References

[CR1] Acin-Perez R, Bayona-Bafaluy MP, Fernandez-Silva P (2004). Respiratory complex III is required to maintain complex I in mammalian mitochondria. Mol Cell.

[CR2] Aeby A, Sznajer Y, Cave H (2007). Cardiofaciocutaneous (CFC) syndrome associated with muscular coenzyme Q10 deficiency. J Inherit Metab Dis.

[CR3] Anderson S, Bankier AT, Barrell BG (1981). Sequence and organization of the human mitochondrial genome. Nature.

[CR4] Antonicka H, Ostergaard E, Sasarman F (2010). Mutations in C12orf65 in patients with encephalomyopathy and a mitochondrial translation defect. Am J Hum Genet.

[CR5] Balreira A, Boczonadi V, Barca E (2014). ANO10 mutations cause ataxia and coenzyme Q10 deficiency. J Neurol.

[CR6] Bannwarth S, Ait-El-Mkadem S, Chaussenot A (2014). A mitochondrial origin for frontotemporal dementia and amyotrophic lateral sclerosis through CHCHD10 involvement. Brain.

[CR7] Bonnen PE, Yarham JW, Besse A (2013). Mutations in FBXL4 cause mitochondrial encephalopathy and a disorder of mitochondrial DNA maintenance. Am J Hum Genet.

[CR8] Bourdon A, Minai L, Serre V (2007). Mutation of RRM2B, encoding p53-controlled ribonucleotide reductase (p53R2), causes severe mitochondrial DNA depletion. Nat Genet.

[CR9] Brea-Calvo G, Haack TB, Karall D (2015). COQ4 Mutations Cause a Broad Spectrum of Mitochondrial Disorders Associated with CoQ10 Deficiency. Am J Hum Genet.

[CR10] Broomfield A, Sweeney MG, Woodward CE, et al (2014) Paediatric single mitochondrial DNA deletion disorders: an overlapping spectrum of disease. J Inherit Metab Dis. doi:10.1007/s10545-014-9778-4 10.1007/s10545-014-9778-4 PMC4432108 25352051

[CR11] Brown GK, Hunt SM, Scholem R (1982). beta-hydroxyisobutyryl coenzyme A deacylase deficiency: a defect in valine metabolism associated with physical malformations. Pediatrics.

[CR12] Bykhovskaya Y, Casas K, Mengesha E, Inbal A, Fischel-Ghodsian N (2004). Missense mutation in pseudouridine synthase 1 (PUS1) causes mitochondrial myopathy and sideroblastic anemia (MLASA). Am J Hum Genet.

[CR13] Cagnoli C, Mariotti C, Taroni F (2006). SCA28, a novel form of autosomal dominant cerebellar ataxia on chromosome 18p11.22-q11.2. Brain.

[CR14] Camaschella C, Campanella A, De Falco L (2007). The human counterpart of zebrafish shiraz shows sideroblastic-like microcytic anemia and iron overload. Blood.

[CR15] Carroll CJ, Isohanni P, Poyhonen R (2013). Whole-exome sequencing identifies a mutation in the mitochondrial ribosome protein MRPL44 to underlie mitochondrial infantile cardiomyopathy. J Med Genet.

[CR16] Casari G, De Fusco M, Ciarmatori S (1998). Spastic paraplegia and OXPHOS impairment caused by mutations in paraplegin, a nuclear-encoded mitochondrial metalloprotease. Cell.

[CR17] Cassereau J, Chevrollier A, Gueguen N (2009). Mitochondrial complex I deficiency in GDAP1-related autosomal dominant Charcot-Marie-Tooth disease (CMT2K). Neurogenetics.

[CR18] Chakraborty PK, Schmitz-Abe K, Kennedy EK (2014). Mutations in TRNT1 cause congenital sideroblastic anemia with immunodeficiency, fevers, and developmental delay (SIFD). Blood.

[CR19] Coenen MJ, Antonicka H, Ugalde C (2004). Mutant mitochondrial elongation factor G1 and combined oxidative phosphorylation deficiency. N Engl J Med.

[CR20] Copeland WC (2008). Inherited mitochondrial diseases of DNA replication. Annu Rev Med.

[CR21] Crosby AH, Patel H, Chioza BA (2010). Defective mitochondrial mRNA maturation is associated with spastic ataxia. Am J Hum Genet.

[CR22] de Keyzer Y, Valayannopoulos V, Benoist JF (2009). Multiple OXPHOS deficiency in the liver, kidney, heart, and skeletal muscle of patients with methylmalonic aciduria and propionic aciduria. Pediatr Res.

[CR23] De Kremer RD, Paschini-Capra A, Bacman S (2001). Barth's syndrome-like disorder: a new phenotype with a maternally inherited A3243G substitution of mitochondrial DNA (MELAS mutation). Am J Med Genet.

[CR24] De Rocco D, Cerqua C, Goffrini P (2014). Mutations of cytochrome c identified in patients with thrombocytopenia THC4 affect both apoptosis and cellular bioenergetics. Biochim Biophys Acta.

[CR25] Desbats MA, Lunardi G, Doimo M, Trevisson E, Salviati L (2014) Genetic bases and clinical manifestations of coenzyme Q (CoQ ) deficiency. J Inherit Metab Dis doi: 10.1007/s10545-014-9749-9 10.1007/s10545-014-9749-9 25091424

[CR26] Deutschmann AJ, Amberger A, Zavadil C (2014). Mutation or knock-down of 17beta-hydroxysteroid dehydrogenase type 10 cause loss of MRPP1 and impaired processing of mitochondrial heavy strand transcripts. Hum Mol Genet.

[CR27] Di Bella D, Lazzaro F, Brusco A (2010). Mutations in the mitochondrial protease gene AFG3L2 cause dominant hereditary ataxia SCA28. Nat Genet.

[CR28] Di Fonzo A, Ronchi D, Lodi T (2009). The mitochondrial disulfide relay system protein GFER is mutated in autosomal-recessive myopathy with cataract and combined respiratory-chain deficiency. Am J Hum Genet.

[CR29] Diaz F, Enriquez JA, Moraes CT (2012). Cells lacking Rieske iron-sulfur protein have a reactive oxygen species-associated decrease in respiratory complexes I and IV. Mol Cell Biol.

[CR30] Diodato D, Ghezzi D, Tiranti V (2014). The Mitochondrial Aminoacyl tRNA Synthetases: Genes and Syndromes. Int J Cell Biol.

[CR31] Dubeau F, De Stefano N, Zifkin BG, Arnold DL, Shoubridge EA (2000). Oxidative phosphorylation defect in the brains of carriers of the tRNAleu(UUR) A3243G mutation in a MELAS pedigree. Ann Neurol.

[CR32] Ferreira C, Thompson R, Vernon H (1993) Barth syndrome. In: Pagon RA, Adam MP, Ardinger HH et al (eds) GeneReviews, University of Washington, Seattle

[CR33] Gai X, Ghezzi D, Johnson MA (2013). Mutations in FBXL4, encoding a mitochondrial protein, cause early-onset mitochondrial encephalomyopathy. Am J Hum Genet.

[CR34] Galmiche L, Serre V, Beinat M (2011). Exome sequencing identifies MRPL3 mutation in mitochondrial cardiomyopathy. Hum Mutat.

[CR35] Garcia-Diaz B, Barros MH, Sanna-Cherchi S (2012). Infantile encephaloneuromyopathy and defective mitochondrial translation are due to a homozygous RMND1 mutation. Am J Hum Genet.

[CR36] Gasparre G, Hervouet E, de Laplanche E (2008). Clonal expansion of mutated mitochondrial DNA is associated with tumor formation and complex I deficiency in the benign renal oncocytoma. Hum Mol Genet.

[CR37] Gellerich FN, Mayr JA, Reuter S, Sperl W, Zierz S (2004). The problem of interlab variation in methods for mitochondrial disease diagnosis: enzymatic measurement of respiratory chain complexes. Mitochondrion.

[CR38] Gempel K, Topaloglu H, Talim B (2007). The myopathic form of coenzyme Q10 deficiency is caused by mutations in the electron-transferring-flavoprotein dehydrogenase (ETFDH) gene. Brain.

[CR39] Ghezzi D, Baruffini E, Haack TB (2012). Mutations of the mitochondrial-tRNA modifier MTO1 cause hypertrophic cardiomyopathy and lactic acidosis. Am J Hum Genet.

[CR40] Gibson K, Halliday JL, Kirby DM, Yaplito-Lee J, Thorburn DR, Boneh A (2008). Mitochondrial oxidative phosphorylation disorders presenting in neonates: clinical manifestations and enzymatic and molecular diagnoses. Pediatrics.

[CR41] Gorman GS, Pfeffer G, Griffin H et al (2014) Clonal Expansion of Secondary Mitochondrial DNA Deletions Associated With Spinocerebellar Ataxia Type 28. JAMA Neurol 72(1):106–111 doi: 10.1001/jamaneurol.2014.1753 10.1001/jamaneurol.2014.1753 25420100

[CR42] Grady JP, Campbell G, Ratnaike T (2014). Disease progression in patients with single, large-scale mitochondrial DNA deletions. Brain.

[CR43] Grau T, Burbulla LF, Engl G (2013). A novel heterozygous OPA3 mutation located in the mitochondrial target sequence results in altered steady-state levels and fragmented mitochondrial network. J Med Genet.

[CR44] Haack TB, Kopajtich R, Freisinger P (2013). ELAC2 mutations cause a mitochondrial RNA processing defect associated with hypertrophic cardiomyopathy. Am J Hum Genet.

[CR45] Haas D, Niklowitz P, Horster F (2009). Coenzyme Q(10) is decreased in fibroblasts of patients with methylmalonic aciduria but not in mevalonic aciduria. J Inherit Metab Dis.

[CR46] Haghighi A, Haack TB, Atiq M (2014). Sengers syndrome: six novel AGK mutations in seven new families and review of the phenotypic and mutational spectrum of 29 patients. Orphanet J Rare Dis.

[CR47] Hallmann K, Zsurka G, Moskau-Hartmann S (2014). A homozygous splice-site mutation in CARS2 is associated with progressive myoclonic epilepsy. Neurology.

[CR48] Hansen JJ, Durr A, Cournu-Rebeix I (2002). Hereditary spastic paraplegia SPG13 is associated with a mutation in the gene encoding the mitochondrial chaperonin Hsp60. Am J Hum Genet.

[CR49] Hildick-Smith GJ, Cooney JD, Garone C (2013). Macrocytic anemia and mitochondriopathy resulting from a defect in sideroflexin 4. Am J Hum Genet.

[CR50] Holt IJ, Jacobs HT (2014). Unique features of DNA replication in mitochondria: a functional and evolutionary perspective. Bioessays.

[CR51] Honzik T, Tesarova M, Magner M (2012). Neonatal onset of mitochondrial disorders in 129 patients: clinical and laboratory characteristics and a new approach to diagnosis. J Inherit Metab Dis.

[CR52] Hudson G, Amati-Bonneau P, Blakely EL (2008). Mutation of OPA1 causes dominant optic atrophy with external ophthalmoplegia, ataxia, deafness and multiple mitochondrial DNA deletions: a novel disorder of mtDNA maintenance. Brain.

[CR53] Janer A, Antonicka H, Lalonde E (2012). An RMND1 Mutation causes encephalopathy associated with multiple oxidative phosphorylation complex deficiencies and a mitochondrial translation defect. Am J Hum Genet.

[CR54] Jenkinson EM, Clayton-Smith J, Mehta S (2012). Perrault syndrome: further evidence for genetic heterogeneity. J Neurol.

[CR55] Jin H, May M, Tranebjaerg L (1996). A novel X-linked gene, DDP, shows mutations in families with deafness (DFN-1), dystonia, mental deficiency and blindness. Nat Genet.

[CR56] Karkucinska-Wieckowska A, Trubicka J, Werner B (2013). Left ventricular noncompaction (LVNC) and low mitochondrial membrane potential are specific for Barth syndrome. J Inherit Metab Dis.

[CR57] Kopajtich R, Nicholls TJ, Rorbach J (2014). Mutations in GTPBP3 cause a mitochondrial translation defect associated with hypertrophic cardiomyopathy, lactic acidosis, and encephalopathy. Am J Hum Genet.

[CR58] Kornblum C, Nicholls TJ, Haack TB (2013). Loss-of-function mutations in MGME1 impair mtDNA replication and cause multisystemic mitochondrial disease. Nat Genet.

[CR59] Kovarova N, Cizkova Vrbacka A, Pecina P (2012). Adaptation of respiratory chain biogenesis to cytochrome c oxidase deficiency caused by SURF1 gene mutations. Biochim Biophys Acta.

[CR60] Leshinsky-Silver E, Malinger G, Ben-Sira L (2011). A large homozygous deletion in the SAMHD1 gene causes atypical Aicardi-Goutieres syndrome associated with mtDNA deletions. Eur J Hum Genet.

[CR61] Liu G, Guo S, Anderson GJ, Camaschella C, Han B, Nie G (2014). Heterozygous missense mutations in the GLRX5 gene cause sideroblastic anemia in a Chinese patient. Blood.

[CR62] Lopez LC, Schuelke M, Quinzii CM (2006). Leigh syndrome with nephropathy and CoQ10 deficiency due to decaprenyl diphosphate synthase subunit 2 (PDSS2) mutations. Am J Hum Genet.

[CR63] Loupatty FJ, Clayton PT, Ruiter JP (2007). Mutations in the gene encoding 3-hydroxyisobutyryl-CoA hydrolase results in progressive infantile neurodegeneration. Am J Hum Genet.

[CR64] Mayr JA (2014). Lipid metabolism in mitochondrial membranes. J Inherit Metab Dis.

[CR65] Mayr JA, Haack TB, Graf E (2012). Lack of the mitochondrial protein acylglycerol kinase causes Sengers syndrome. Am J Hum Genet.

[CR66] Mayr JA, Feichtinger RG, Tort F, Ribes A, Sperl W (2014). Lipoic acid biosynthesis defects. J Inherit Metab Dis.

[CR67] McKenzie M, Lazarou M, Thorburn DR, Ryan MT (2006). Mitochondrial respiratory chain supercomplexes are destabilized in Barth Syndrome patients. J Mol Biol.

[CR68] Melchionda L, Haack TB, Hardy S (2014). Mutations in APOPT1, encoding a mitochondrial protein, cause cavitating leukoencephalopathy with cytochrome c oxidase deficiency. Am J Hum Genet.

[CR69] Miller C, Saada A, Shaul N (2004). Defective mitochondrial translation caused by a ribosomal protein (MRPS16) mutation. Ann Neurol.

[CR70] Miyake N, Yano S, Sakai C (2013). Mitochondrial complex III deficiency caused by a homozygous UQCRC2 mutation presenting with neonatal-onset recurrent metabolic decompensation. Hum Mutat.

[CR71] Morino H, Pierce SB, Matsuda Y (2014). Mutations in Twinkle primase-helicase cause Perrault syndrome with neurologic features. Neurology.

[CR72] Morison IM, Cramer Borde EM, Cheesman EJ (2008). A mutation of human cytochrome c enhances the intrinsic apoptotic pathway but causes only thrombocytopenia. Nat Genet.

[CR73] Nesbitt V, Pitceathly RD, Turnbull DM (2013). The UK MRC Mitochondrial Disease Patient Cohort Study: clinical phenotypes associated with the m.3243A > G mutation–implications for diagnosis and management. J Neurol Neurosurg Psychiatry.

[CR74] Neupert W, Herrmann JM (2007). Translocation of proteins into mitochondria. Annu Rev Biochem.

[CR75] Newman WG, Friedman TB, Conway GS (1993) Perrault Syndrome. In: Pagon RA, Adam MP, Ardinger HH et al (eds) GeneReviews, Seattle (WA) 25254289

[CR76] O'Toole JF (2014). Renal manifestations of genetic mitochondrial disease. Int J Nephrol Renovasc Dis.

[CR77] O'Toole JF, Liu Y, Davis EE (2010). Individuals with mutations in XPNPEP3, which encodes a mitochondrial protein, develop a nephronophthisis-like nephropathy. J Clin Invest.

[CR78] Pfeffer G, Gorman GS, Griffin H (2014). Mutations in the SPG7 gene cause chronic progressive external ophthalmoplegia through disordered mitochondrial DNA maintenance. Brain.

[CR79] Prasad C, Melancon SB, Rupar CA (2013). Exome sequencing reveals a homozygous mutation in TWINKLE as the cause of multisystemic failure including renal tubulopathy in three siblings. Mol Genet Metab.

[CR80] Puy H, Gouya L, Deybach JC (2010). Porphyrias. Lancet.

[CR81] Quinzii CM, Kattah AG, Naini A (2005). Coenzyme Q deficiency and cerebellar ataxia associated with an aprataxin mutation. Neurology.

[CR82] Quinzii CM, Emmanuele V, Hirano M (2014). Clinical presentations of coenzyme Q10 deficiency syndrome. Mol Syndromol.

[CR83] Rackham O, Filipovska A (2014). Supernumerary proteins of mitochondrial ribosomes. Biochim Biophys Acta.

[CR84] Rahman S (2013). Gastrointestinal and hepatic manifestations of mitochondrial disorders. J Inherit Metab Dis.

[CR85] Richter-Dennerlein R, Korwitz A, Haag M (2014). DNAJC19, a mitochondrial cochaperone associated with cardiomyopathy, forms a complex with prohibitins to regulate cardiolipin remodeling. Cell Metab.

[CR86] Riley LG, Cooper S, Hickey P (2010). Mutation of the mitochondrial tyrosyl-tRNA synthetase gene, YARS2, causes myopathy, lactic acidosis, and sideroblastic anemia–MLASA syndrome. Am J Hum Genet.

[CR87] Ronchi D, Di Fonzo A, Lin W (2013). Mutations in DNA2 link progressive myopathy to mitochondrial DNA instability. Am J Hum Genet.

[CR88] Rouzier C, Bannwarth S, Chaussenot A (2012). The MFN2 gene is responsible for mitochondrial DNA instability and optic atrophy 'plus' phenotype. Brain.

[CR89] Ruiz-Pesini E, Lott MT, Procaccio V (2007). An enhanced MITOMAP with a global mtDNA mutational phylogeny. Nucleic Acids Res.

[CR90] Saada A, Shaag A, Arnon S (2007). Antenatal mitochondrial disease caused by mitochondrial ribosomal protein (MRPS22) mutation. J Med Genet.

[CR91] Sakai C, Yamaguchi S, Sasaki M, Miyamoto Y, Matsushima Y, Goto YI (2014) ECHS1 mutations cause combined respiratory chain deficiency resulting Leigh syndrome. Hum Mutat 36(2):232–239 10.1002/humu.22730 25393721

[CR92] Santorelli FM, Tanji K, Sano M (1997). Maternally inherited encephalopathy associated with a single-base insertion in the mitochondrial tRNATrp gene. Ann Neurol.

[CR93] Sarkar D, Fisher PB (2006). Human polynucleotide phosphorylase (hPNPase old-35): an RNA degradation enzyme with pleiotrophic biological effects. Cell Cycle.

[CR94] Sasarman F, Brunel-Guitton C, Antonicka H, Wai T, Shoubridge EA (2010). LRPPRC and SLIRP interact in a ribonucleoprotein complex that regulates posttranscriptional gene expression in mitochondria. Mol Biol Cell.

[CR95] Scaglia F, Towbin JA, Craigen WJ (2004). Clinical spectrum, morbidity, and mortality in 113 pediatric patients with mitochondrial disease. Pediatrics.

[CR96] Schagger H, Pfeiffer K (2000). Supercomplexes in the respiratory chains of yeast and mammalian mitochondria. EMBO J.

[CR97] Schon EA, DiMauro S, Hirano M (2012). Human mitochondrial DNA: roles of inherited and somatic mutations. Nat Rev Genet.

[CR98] Schrader M, Bonekamp NA, Islinger M (2012). Fission and proliferation of peroxisomes. Biochim Biophys Acta.

[CR99] Schwartzentruber J, Buhas D, Majewski J et al (2014) Mutation in the nuclear-encoded mitochondrial isoleucyl-tRNA synthetase IARS2 in patients with cataracts, growth hormone deficiency with short stature, partial sensorineural deafness, and peripheral neuropathy or with Leigh syndrome. Hum Mutat 35:1285–1289 10.1002/humu.22629 25130867

[CR100] Serre V, Rozanska A, Beinat M (2013). Mutations in mitochondrial ribosomal protein MRPL12 leads to growth retardation, neurological deterioration and mitochondrial translation deficiency. Biochim Biophys Acta.

[CR101] Shamseldin HE, Alshammari M, Al-Sheddi T (2012). Genomic analysis of mitochondrial diseases in a consanguineous population reveals novel candidate disease genes. J Med Genet.

[CR102] Smeitink JA, Elpeleg O, Antonicka H (2006). Distinct clinical phenotypes associated with a mutation in the mitochondrial translation elongation factor EFTs. Am J Hum Genet.

[CR103] Stehling O, Wilbrecht C, Lill R (2014). Mitochondrial iron-sulfur protein biogenesis and human disease. Biochimie.

[CR104] Suzuki T, Nagao A (2011). Human mitochondrial tRNAs: biogenesis, function, structural aspects, and diseases. Annu Rev Genet.

[CR105] Taylor RW, Pyle A, Griffin H (2014). Use of whole-exome sequencing to determine the genetic basis of multiple mitochondrial respiratory chain complex deficiencies. JAMA.

[CR106] Tchikviladze M, Gilleron M, Maisonobe T, et al (2014) A diagnostic flow chart for POLG-related diseases based on signs sensitivity and specificity. J Neurol Neurosurg Psychiatry. doi:10.1136/jnnp-2013-306799 10.1136/jnnp-2013-306799 25118206

[CR107] Thorburn DR, Rahman S (1993) Mitochondrial DNA-associated Leigh syndrome and NARP. In: Pagon RA, Adam MP, Ardinger HH et al (eds) GeneReviews, University of Washington, Seattle (WA) 20301352

[CR108] Tiranti V, Viscomi C, Hildebrandt T (2009). Loss of ETHE1, a mitochondrial dioxygenase, causes fatal sulfide toxicity in ethylmalonic encephalopathy. Nat Med.

[CR109] Tucker EJ, Hershman SG, Kohrer C (2011). Mutations in MTFMT underlie a human disorder of formylation causing impaired mitochondrial translation. Cell Metab.

[CR110] Valente L, Tiranti V, Marsano RM (2007). Infantile encephalopathy and defective mitochondrial DNA translation in patients with mutations of mitochondrial elongation factors EFG1 and EFTu. Am J Hum Genet.

[CR111] van Rahden VA, Rau I, Fuchs S (2014). Clinical spectrum of females with HCCS mutation: from no clinical signs to a neonatal lethal form of the microphthalmia with linear skin defects (MLS) syndrome. Orphanet J Rare Dis.

[CR112] Vanlander AV, Menten B, Smet J et al (2014) Two siblings with homozygous pathogenic splice site variant in mitochondrial asparaginyl-tRNA synthetase (NARS2). Hum Mutat 36(2):222–231 doi: 10.1002/humu.22728 10.1002/humu.22728 25385316

[CR113] Vedrenne V, Galmiche L, Chretien D, de Lonlay P, Munnich A, Rotig A (2012). Mutation in the mitochondrial translation elongation factor EFTs results in severe infantile liver failure. J Hepatol.

[CR114] Vedrenne V, Gowher A, De Lonlay P (2012). Mutation in PNPT1, which encodes a polyribonucleotide nucleotidyltransferase, impairs RNA import into mitochondria and causes respiratory-chain deficiency. Am J Hum Genet.

[CR115] Vogtle FN, Wortelkamp S, Zahedi RP (2009). Global analysis of the mitochondrial N-proteome identifies a processing peptidase critical for protein stability. Cell.

[CR116] Wang G, Shimada E, Koehler CM, Teitell MA (2012). PNPASE and RNA trafficking into mitochondria. Biochim Biophys Acta.

[CR117] Waterham HR, Koster J, van Roermund CW, Mooyer PA, Wanders RJ, Leonard JV (2007). A lethal defect of mitochondrial and peroxisomal fission. N Engl J Med.

[CR118] Wedding IM, Koht J, Tran GT (2014). Spastic paraplegia type 7 is associated with multiple mitochondrial DNA deletions. PLoS One.

[CR119] Wittig I, Schagger H (2008). Structural organization of mitochondrial ATP synthase. Biochim Biophys Acta.

[CR120] Wortmann SB, De Brouwer APM, Wevers RA, Morava E (1993) MEGDEL syndrome. In: Pagon RA, Adam MP, Ardinger HH et al (eds) GeneReviews(R), University of Washington, Seattle (WA)

[CR121] Wortmann SB, Vaz FM, Gardeitchik T (2012). Mutations in the phospholipid remodeling gene SERAC1 impair mitochondrial function and intracellular cholesterol trafficking and cause dystonia and deafness. Nat Genet.

[CR122] Wortmann SB, Kluijtmans LA, Rodenburg RJ (2013). 3-Methylglutaconic aciduria–lessons from 50 genes and 977 patients. J Inherit Metab Dis.

[CR123] Yano H, Baranov SV, Baranova OV (2014). Inhibition of mitochondrial protein import by mutant huntingtin. Nat Neurosci.

[CR124] Yarham JW, Lamichhane TN, Pyle A (2014). Defective i6A37 modification of mitochondrial and cytosolic tRNAs results from pathogenic mutations in TRIT1 and its substrate tRNA. PLoS Genet.

[CR125] Zeharia A, Shaag A, Pappo O (2009). Acute infantile liver failure due to mutations in the TRMU gene. Am J Hum Genet.

[CR126] Zierz CM, Joshi PR, Zierz S (2014) Frequencies of myohistological mitochondrial changes in patients with mitochondrial DNA deletions and the common m.3243A > G point mutation. Neuropathology. doi:10.1111/neup.12173 10.1111/neup.12173 25378026

[CR127] Zimmermann FA, Mayr JA, Feichtinger R (2011). Respiratory chain complex I is a mitochondrial tumor suppressor of oncocytic tumors. Front Biosci (Elite Ed).

